# Zero inflated high dimensional compositional data with DeepInsight

**DOI:** 10.1371/journal.pone.0320832

**Published:** 2025-04-16

**Authors:** Jeseok Lee, Byungwon Kim

**Affiliations:** Department of Statistics, Kyungpook National University, Daegu, South Korea; Bournemouth University, UNITED KINGDOM OF GREAT BRITAIN AND NORTHERN IRELAND

## Abstract

Through the Human Microbiome Project, research on human-associated microbiomes has been conducted in various fields. New sequencing techniques such as Next Generation Sequencing (NGS) and High-Throughput Sequencing (HTS) have enabled the inclusion of a wide range of features of the microbiome. These advancements have also contributed to the development of numerical proxies like Operational Taxonomic Units (OTUs) and Amplicon Sequence Variants (ASVs). Studies involving such microbiome data often encounter zero-inflated and high-dimensional problems. Based on the need to address these two issues and the recent emphasis on compositional interpretation of microbiome data, we conducted our research. To solve the zero-inflated problem in compositional microbiome data, we transformed the data onto the surface of the hypersphere using a square root transformation. Then, to solve the high-dimensional problem, we modified DeepInsight, an image-generating method using Convolutional Neural Networks (CNNs), to fit the hypersphere space. Furthermore, to resolve the common issue of distinguishing between true zero values and fake zero values in zero-inflated images, we added a small value to the true zero values. We validated our approach using pediatric inflammatory bowel disease (IBD) fecal sample data and achieved an area under the curve (AUC) value of 0.847, which is higher than the previous study’s result of 0.83.

## Introduction

The Human Microbiome Project has significantly advanced our understanding of the human microbiome [1], enabling comprehensive studies on its role in various diseases such as cancer [2–4], Crohn’s disease [5,6], and obesity [7,8]. By utilizing numerical proxies such as Operational Taxonomic Units (OTUs) and Amplicon Sequence Variants (ASVs), researchers have been able to quantify microbiome data more effectively, facilitating more natural data representations [9–12]. Consequently, a wide range of statistical and machine learning techniques have been developed and applied to analyze these microbiome datasets. Although microbiome data are fundamentally count data, limitations of sequencing technologies like 16S rRNA sequencing constrain the total sequencing depth, leading to the interpretation of microbiome data as compositional data [13].

Compositional data is a type of non-Euclidean data in which all components are positive and their sum is fixed at a constant value. Due to this fixed sum, compositional data does not have full rank. Therefore, compositional data is defined within a specialized space known as the simplex [14]. This non-Euclidean sample space poses significant challenges for the direct application of traditional statistical methods, which typically assume Euclidean space properties. To overcome these challenges, log-ratio transformations have become the most widely used approach for mapping compositional data into Euclidean space [15,16]. However, these transformations are unable to properly handle the zero-inflated problem commonly encountered in microbiome data. The zero-inflated problem occurs when datasets contain an excessive number of zeros that cannot be explained by typical statistical distributions [17]. In microbiome data, this often happens due to the absence of certain microbial taxa in some samples, leading to many zero values. These zeros can be either structural, where a particular taxon is truly absent, or sampling-related, where the taxon is present but undetected due to limitations in sequencing depth or sampling [18]. This poses challenges for standard modeling approaches, which do not account for the dual nature of these zeros. To address the zero-inflated problem, methods such as Bayesian-Multiplicative replacement, which is implemented in the cmultRepl function of the R package Zcompositions have been used to replace zero values [19]. Despite their utility, zero-replacement methods can cause distortions, prompting the exploration of alternative approaches that directly handle zero values without replacement [20–24].

The square-root transformation provides an effective solution by mapping compositional data onto the surface of the hypersphere, enabling the use of probability distributions defined on the hypersphere, such as the Kent distribution [25–27]. Furthermore, dimension reduction is also possible using Principal Geodesic Analysis (PGA). PGA extends Principal Component Analysis (PCA) by incorporating the intrinsic geometry of Riemannian manifolds. PGA identifies the main modes of variation along geodesics, the shortest paths on the manifold, providing a more accurate representation of the data’s variability on curved surfaces [28,29]. The detailed methodology of applying PGA in this study is provided in the Materials and Methods section. Based on the square-root transformation, this study analyzes zero-inflated compositional data, preserving the integrity of the original data while effectively handling exactly zero values.

New sequencing techniques, known as Next Generation Sequencing (NGS) or High-Throughput Sequencing (HTS), have led to a rapid increase in both the volume and complexity of microbiome data. High-dimensional microbiome datasets contain a wide range of features, including genomes, transcriptomes, proteomes, and metagenomes, often outnumbering the available biological samples. This high dimensionality poses significant challenges for data analysis, including heightened computational demands, increased risk of overfitting, and difficulties in interpreting results [30–32]. Image generating methods, such as DeepInsight [33] and Image Generator for Tabular Data (IGTD) [34], have been proposed to address high-dimensional problems by leveraging powerful deep learning models like Convolutional Neural Networks (CNNs). These methods convert non-image data into image formats based on data through techniques such as dimension reduction, pairwise correlation matrices, and clustering, enabling CNNs to effectively analyze complex patterns and relationships within the data. This approach is particularly beneficial for managing unstructured and high-dimensional datasets, including those found in microbiome and voice data analyses.

Segmentation, the process of separating the foreground from the background in image analysis, is a fundamental task that significantly impacts the accuracy and effectiveness of image interpretation. In deep learning-based image analysis, methods such as U-Net and SegNet have demonstrated remarkable performance and have garnered considerable attention in the field. These segmentation techniques play a critical role in clarifying the boundaries between the foreground and background, thereby facilitating the learning of image features by CNNs. By accurately delineating different regions within an image, segmentation algorithms enable models to focus on relevant patterns and structures, improving overall performance in tasks like object recognition and scene understanding. In our study, we use image datasets generated by DeepInsight, which transforms non-image data into image formats based on the data values themselves. This data-driven approach can present challenges when dealing with zero-inflated data. To address this issue, we propose adding small values to the true zero values to distinguish between true zero values associated with the foreground and fake zero values associated with the background. The method for adding these values is described in the Materials and Methods section.

In this study, we conducted an analysis of zero-inflated high-dimensional compositional data. First, to address the zero-inflated problem, we transformed the data onto the surface of the hypersphere using the square-root transformation. Next, we modified the DeepInsight algorithm to suit the hypersphere and applied it for analysis. In this process, we added a small value to distinguish between true zero values and fake zero values in the zero-inflated image. Finally, we validated the results using the fecal sample data employed by Papa et al.(2012) [35]. This dataset was designed for the classification of Inflammatory Bowel Disease (IBD), such as Crohn’s disease. Using this dataset, we compared the performance of our modified DeepInsight with that of previous studies.

## Materials and methods

### Compositional data

Compositional data is a type of non-Euclidean spatial data consisting of positive components that sum to a constant value. Although the sum of the components is not inherently restricted, it is commonly normalized to 1 for simplicity, effectively treating the sample space as a simplex [14]. When considering a *d* - dimensional compositional vector **x**, it is expressed as $x\in S^{d-1}$, where $S^{d-1}$ denotes the unit simplex defined as follows:


$$\begin{array}{ccc} S^{d-1}=\left\{x=[x_{1},x_{2},\cdots \;,x_{d}]\in {\mathbb{R}}^{d}|\;x_{i}>0,i=1,2,\cdots \;,d;\overset{d}{\underset{i=1}{\sum }}x_{i}=1\right\}. & & \end{array}$$ (1)

### Square root transformation

The square-root transformation, a type of power transformation, maps a compositional vector $x=(x_{1},x_{2},\ldots ,x_{d})\in S^{d-1}$ (See Eq 1) onto the surface of the (*d* – 1) - dimensional unit hypersphere ${\mathbb{C}}^{d-1}$. The transformation is defined as follows:


$$y=T(x)=(\sqrt{x_{1}},\sqrt{x_{2}},\ldots ,\sqrt{x_{d}}).$$

The square-root transformation offers several advantages. It allows direct handling of zeros; unlike log-ratio transformations, which are undefined for zero values and require substitution or adjustment, the square-root transformation naturally accommodates zeros. By mapping data onto the surface of the hypersphere, we can apply statistical methods developed for directional data, including using probability distributions like the von Mises–Fisher and Kent distributions to model and analyze the data’s directional characteristics [26]. Techniques such as spherical regression, clustering on the sphere, and spherical harmonic analysis become applicable, providing deeper insights into the data structure.

### DeepInsight

DeepInsight, proposed by Sharma et al.(2019) [33], is a methodology for converting non-image data into image format to apply CNNs. It offers a general approach applicable to various irregular and non-structured data such as genomics, transcriptomics, methylation, mutations, text, spoken words, and financial data.

**Fig 1:**
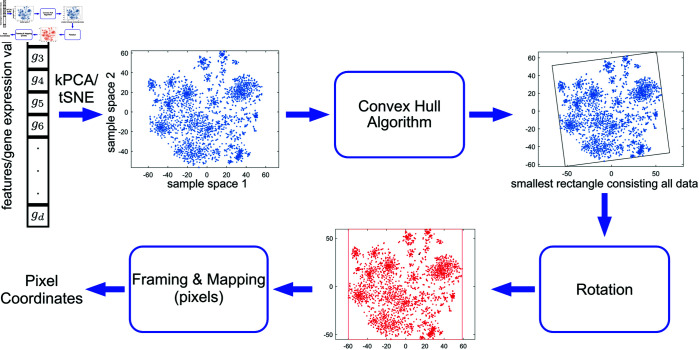
DeepInsight pipeline. This is the DeepInsight pipeline provided by Sharma et al. (2019). It creates representative images through dimension reduction and optimizes the images using the Convex Hull algorithm. Subsequently, it generates image-specific differentiation through feature matrices and normalization, and creates images by mapping them to pixels.

Algorithm 1 provides a concise overview of the DeepInsight algorithm, and Fig 1 is the pipeline presented by Sharma et al. (2019) [33]. The process begins by transposing the input data matrix *X* into a feature-focused matrix *G*, which allows the algorithm to concentrate on relationships between features rather than samples.

**Algorithm 1: DeepInsight pipeline with convex hull.**

**Require:** Training set $X=\{x_{1},x_{2},...,x_{n}\}$ with *d* features, where $x_{i}\in {\mathbb{R}}^{d}$

**Ensure:** Image representation of non-image data for CNNs input

1: **Step 1: Transpose Data Matrix**

2: Define feature set $G=\{g_{1},g_{2},...,g_{h}\}$ by transposing *X*

3: **Step 2: Dimensionality Reduction**

4: Apply dimensionality reduction (e.g., t-SNE, kernel PCA) on *G* to obtain 2D coordinates

5: **Step 3: Feature Location Mapping & Rearrangement**

6: Map the 2D coordinates to their corresponding pixel locations on a grid, and then perform rearrangement through clustering

7: **Step 4: Apply Convex Hull Algorithm**

8: Apply Convex Hull algorithm to find the smallest bounding polygon for the feature locations

9: **Step 5: Rotate or Adjust the Grid**

10: Rotate or adjust the grid to fit the CNNs input format

11: **Step 6: Feature Normalization**

12: Normalize feature values using one of the following methods:

13: (a) Independent normalization: Normalize each feature independently by its own minimum and maximum values

14: (b) Topology-preserving normalization: Normalize all features using a single global maximum value from the training set

15: Select the normalization method that produces the lowest validation error

16: **Step 7: Final Image Generation**

17: Create the final image with the pixel values representing the normalized features

**Fig 2:**
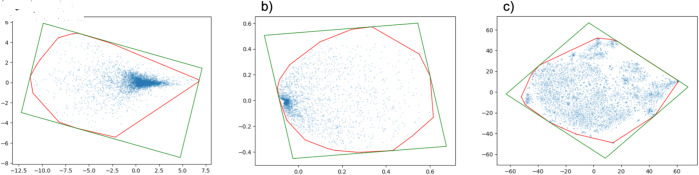
DeepInsight example. Representative images from The Cancer Genome Atlas Program RNA data. a) Process using PCA, b) Process using kernel PCA, and c) Process using t-SNE. Using the Convex Hull algorithm, the red boundary represents the smallest polygon that encloses the corresponding pixels, while the green boundary denotes the smallest rectangle containing them.

Next, dimension reduction methods such as t-SNE or kernel PCA are applied to project the high-dimensional features onto a 2D plane. These 2D coordinates are then mapped to their corresponding pixel locations on a grid, which serves as the basis for the image representation. Following this, clustering techniques are employed to rearrange the pixels based on their spatial relationships. Through this clustering and rearrangement process, the inherent features and hidden patterns within the image can be effectively identified. For example, Fig 2 illustrates representative images generated from The Cancer Genome Atlas Program (TCGA) RNA data. Fig 2a) Process using PCA, Fig 2b) Process using kernel PCA, and Fig 2c) Process using t-SNE. As illustrated in the figure, significant differences in the images emerge depending on the dimension reduction method, affecting the rearrangement and corresponding pixel locations. To optimize for CNNs, the Convex Hull algorithm is used to define the smallest bounding polygon around the feature points, optimizing the space for the feature layout.

**Fig 3:**
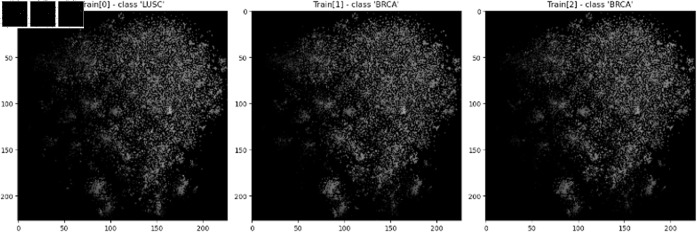
Characteristic image. A characteristic image generated from The Cancer Genome Atlas Program RNA data using t-SNE-based DeepInsight for CNNs training.

The grid is then rotated or adjusted to fit the expected input dimensions of CNNs model. Feature values are normalized using either independent normalization (each feature is normalized by its own min/max values) or topology-preserving normalization (a single global maximum from the entire dataset). The method that results in the lowest validation error is selected. Finally, the normalized feature values are used to generate an image that can be processed by CNNs. Fig 3 is sample images that will be used for CNNs training. Through this process, the DeepInsight demonstrates strengths in classification compared to traditional methods.

### Principal geodesic analysis

PGA is introduced to address the limitations of PCA when dealing with data on Riemannian manifolds such as the hypersphere, which are curved, non-linear spaces. In the context of the hypersphere, data points reside on a curved surface where straight lines are replaced by geodesics, the shortest paths along the sphere’s surface that respect its curvature. PCA is hard to capture the intrinsic geometry of the surface of the hypersphere because it relies on linear approximations and Euclidean distances, which are not suitable for spherical data. PGA overcomes this limitation by utilizing geodesics to define principal directions on the surface of the hypersphere, effectively accounting for its curvature and providing a more accurate analysis of data variability.

When considering the *d* - dimensional unit hypersphere ${\mathbb{C}}^{d-1}$, the point *p* is the Fréchet mean. The Fréchet mean is the point that minimizes the sum of squared geodesic distances to all data points and is used as the reference point for PGA. Typically, a rotation transformation is applied to set this reference point at  ( 0 , 0 , ⋯ , 1 ) .

PGA aims to project the data onto the tangent space $T_{p}$ at the point *p*. Logarithmic map enables projection onto the tangent space, and its definition for $X=(x_{1},x_{2},\cdots \;,x_{d})\in {\mathbb{C}}^{d-1}$ is as follows:


Logp(X)=𝜃sin ⁡ 𝜃⋅(x1,x2,⋯,xd−1),where 𝜃= arccos ⁡ (xd).

This mapping allows the transformation of data from the curved directional space into a flat Euclidean tangent space, enabling the application of PCA. By mapping the data from the manifold onto the tangent space using the logarithmic map, PGA performs PCA in the tangent space to identify principal directions. These directions are then mapped back onto the manifold using the Exponential map, ensuring that the analysis respects the manifold’s curvature.

**Fig 4:**
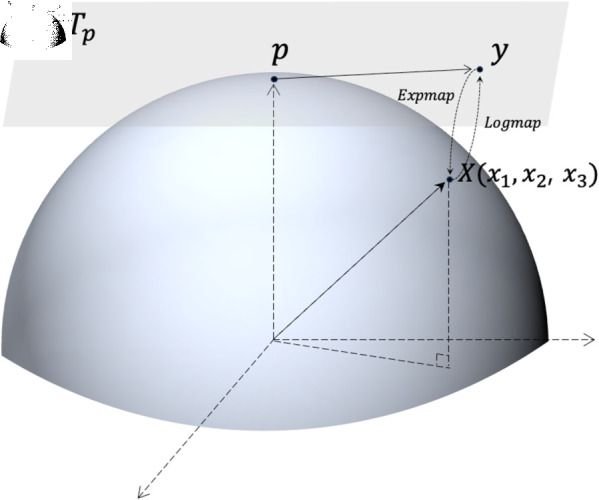
Geodesic PCA. A data point *X* on the 3-dimensional hypersphere ${\mathbb{C}}^{2}$ is mapped to the tangent space $T_{p}$ at the Fréchet mean *p* via the Logarithmic map along the geodesic path. This projection flattens the manifold while preserving the principal directions of variation, enabling the application of PCA in curved spaces.

Additionally, Fig 4 is an example of PGA on the 3-dimensional hypersphere ${\mathbb{C}}^{2}$. The Python geomstats package implements this methodology in code, and we utilized it in our study.

### Segmentation

**Fig 5:**
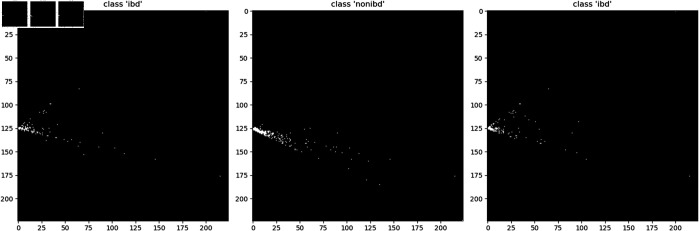
Different structure. To visualize the variation in the overall structure of zero-inflated sample images, we assigned maximum values to the corresponding pixels based on cross-sectional images generated using PCA.

Segmentation enhances image analysis performance by clearly identifying the features or shapes of the foreground through the distinction between the foreground and background. In the case of the existing DeepInsight method, the foreground of the generated images is based on the characteristics of the data, and the shapes are created through dimension reduction, resulting in all images having identical forms. Therefore, the necessity of segmentation was not prominently highlighted. However, when analyzing zero-inflated data using DeepInsight, the color values of the corresponding pixels are determined based on the original data values. If the original value is exactly zero, the pixel color is exactly the same as the background. This causes the shapes of the sample images to be inconsistent, which adversely affects the learning process. An example of this phenomenon is shown in Fig 5.

**Fig 6:**
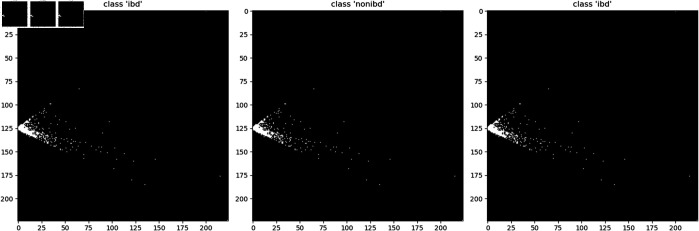
Same structure. To visualize the variation in the overall structure of zero-inflated sample images after adding a small tolerance at corresponding pixels, then we assigned maximum values to the corresponding pixels based on cross-sectional images generated using PCA.

To address this issue, we added a small value to the corresponding pixels generated through DeepInsight, thereby standardizing the shapes of all images as shown in Fig 6.

## Results

### Real data

We analyze the Pediatric Inflammatory Bowel Disease (IBD) dataset from Papa et al.(2012) [35] as a real data example. This dataset consists of 16S rRNA sequencing of fecal samples, from 91 children and young adults who were treated in the gastroenterology program at Children’s Hospital in Boston, USA, including 24 positive cases diagnosed with IBD and 67 negative controls. It contains 36,349 columns, each representing OTUs value, and approximately 98% of the data entries are exactly zero values. The IBD dataset consists of count data collected through 16S rRNA sequencing, allowing it to be interpreted as compositional data. To convert it into a compositional format, the data are divided by the total sum. Subsequently, a square root transformation is applied to project the data onto the surface of a hypersphere.

**Fig 7:**
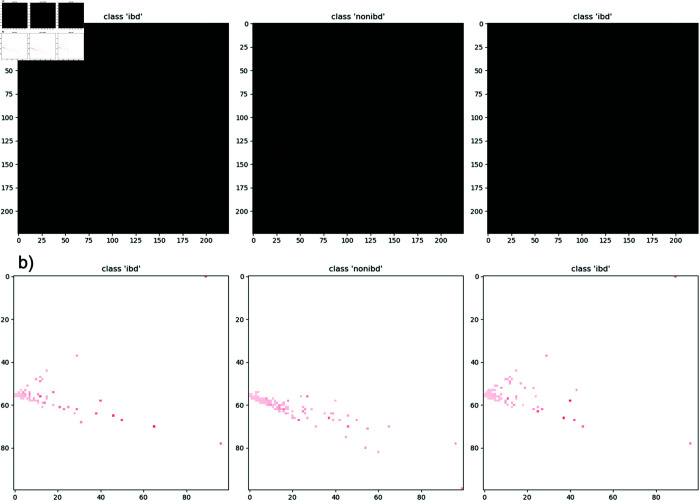
Generated image samples. a) is the image actually used for CNNs training. b) is a rescaled version of the Train Plot, adjusted for visual clarity because the original Train Plot was too dark and not visually appealing; this image was not used for actual training.

### Classification performance

**Algorithm 2: Classification pipeline.**

1: **Step 1: Convert to Compositional Data**

2: Normalize each sample by dividing feature values by the total sum to transform raw count data into compositional data.

3: **Step 2: Square Root Transformation to Directional Space**

4: Apply square root transformation to map the compositional data onto the directional space.

5: **Step 3: Project to Tangent Space using PGA**

6: Perform Principal Geodesic Analysis (PGA) to project points in directional space onto tangent space.

7: **Step 4: Apply DeepInsight for Image Generation**

8: Execute DeepInsight to convert tangent space data into images. In Modified DeepInsight, apply Segmentation by adding a small constant to the corresponding pixel values.

9: **Step 5: Train CNNs and Evaluate Results**

10: Train a CNNs model on the generated images and analyze the classification results

Our modified DeepInsight model focuses on two main objectives. First, DeepInsight is adapted to effectively handle data on the surface of a hypersphere. Second, it involves implementing segmentation to address the issue where background and foreground are treated identically at exact zero values. To achieve these objectives, PGA is employed within the DeepInsight process. Subsequently, segmentation is implemented by adding small values to the positions of the corresponding pixels. As a control group, we also evaluated the original DeepInsight model, which does not apply segmentation to the IBD dataset. After the sample images generated we used ResNet50 for CNNs architecture and AdamW as the optimizer. The learning rate and weight decay were tuned using Optuna, an automated optimization software [36], to maximize the Area Under the Curve (AUC). The learning rate and weight decay were tuned using Optuna, an automated optimization software [36]. The learning rate range was set to [$1\times 10^{-6},1\times 10^{-2}$], while the weight decay range was set to ($1\times 10^{-6},1\times 10^{-2}$). A log-scale search was applied to ensure an even distribution of sampled values across different magnitudes. The input image size was set to 224×224 pixels to match the input requirements of ResNet50. Fig 7a shows the image sample actually used for CNNs training, while Fig 7b shows a rescaled and resized version adjusted to improve visibility for the human eye. Note that the rescaled image was not used for training. The final performance was evaluated by calculating the AUC using 10-fold cross-validation. Algorithm 2 provides a concise representation of our sequential process.

As shown in Table 1, we conducted hyperparameters tuning over 1,000 trials for both the Modified DeepInsight and original DeepInsight using Optuna, selecting the maximum AUC value obtained as the final result for each model. The Modified DeepInsight achieved the performance of 0.847 AUC, exceeding the average AUC of 0.83 reported by Papa et al. (2012) [35], who analyzed fecal samples using Synthetic Learning in Microbial Ecology (SLiME) under three repeated 10-fold cross-validation. Additionally, we observed that the Original DeepInsight achieved the AUC of 0.817, indicating a notably lower performance compared to the Modified DeepInsight. Through these results obtained from real data, we confirmed the applicability of the DeepInsight to zero-inflated high-dimensional compositional data and emphasized the necessity of distinguishing between true zero values and fake zero values.

**Table 1:** Compare classification performance. Papa et al. (2012) applied sequencing data to supervised learning classification algorithms using a software pipeline called Synthetic Learning in Microbial Ecology (SLiME), which utilizes relevant metadata as classification labels. They achieved an average AUC of 0.83 on fecal samples over three repeated 10-fold cross-validation. We trained both the Modified DeepInsight and the Original DeepInsight models 1,000 trials using Optuna and selected the maximum AUC value as the final result.

	SLiME	Modified DeepInsight	Original DeepInsight
*AUC*	0.83	0.847	0.817

## Discussion

In this study, we reconfirmed the DeepInsight continues to demonstrate effective performance on unstructured high-dimensional data. Specifically, we observed that the issue of segmentation, which arises during the process of converting zero-inflated microbiome data into images, also occurs in conventional image analysis. To address this problem, we proposed the Modified DeepInsight that applies a simple method of adding a small constant to the data, achieving performance improvement.

Although the dataset size was small for deep learning, the Modified DeepInsight showed a slight improvement in the AUC compared to SLiME. This demonstrates that deep learning can still be effective with small datasets when appropriate preprocessing and model modifications are applied. Due to methodological differences from previous studies, we could not perform a performance comparison under completely identical conditions, but our results support the validity of DeepInsight. Notably, it is significant that we achieved the higher AUC than the value reported in the past study.

Furthermore, since we confirmed performance improvement with just the simple method of adding a small constant, we believe that developing more sophisticated zero-value handling techniques could lead to additional performance enhancements. Additionally, after transitioning to Tangent Space, we anticipate that employing more advanced dimension reduction methods beyond the PCA we used could further improve performance.

For future research, we aim to enhance data diversity by incorporating raw microbiome data, which we could not address in this study due to technical constraints, through collaboration with domain experts. Moreover, we seek to further explore the applicability of our approach to a broader range of zero-inflated high-dimensional datasets.

## References

[1] Human Microbiome Project Consortium. Structure, function and diversity of the healthy human microbiome. Nature 2012;486(7402):207–14. doi: 10.1038/nature11234 22699609 PMC3564958

[2] Helmink BA, Khan MAW, Hermann A, Gopalakrishnan V, Wargo JA. The microbiome, cancer, and cancer therapy. Nat Med 2019;25(3):377–88. doi: 10.1038/s41591-019-0377-7 30842679

[3] Schwabe RF, Jobin C. The microbiome and cancer. Nat Rev Cancer 2013;13(11):800–12. doi: 10.1038/nrc3610 24132111 PMC3986062

[4] Sepich-Poore GD, Zitvogel L, Straussman R, Hasty J, Wargo JA, Knight R. The microbiome and human cancer. Science. 2021;371(6536):eabc4552. doi: 10.1126/science.abc4552 33766858 PMC8767999

[5] Gevers D, Kugathasan S, Denson LA, Vázquez-Baeza Y, Van Treuren W, Ren B, et al. The treatment-naive microbiome in new-onset Crohn’s disease. Cell Host Microbe 2014;15(3):382–92. doi: 10.1016/j.chom.2014.02.005 24629344 PMC4059512

[6] Halfvarson J, Brislawn CJ, Lamendella R, Vázquez-Baeza Y, Walters WA, Bramer LM, et al. Dynamics of the human gut microbiome in inflammatory bowel disease. Nat Microbiol. 2017;2:17004. doi: 10.1038/nmicrobiol.2017.4 28191884 PMC5319707

[7] Ley RE. Obesity and the human microbiome. Curr Opin Gastroenterol 2010;26(1):5–11. doi: 10.1097/MOG.0b013e328333d751 19901833

[8] Maruvada P, Leone V, Kaplan LM, Chang EB. The human microbiome and obesity: Moving beyond associations. Cell Host Microbe 2017;22(5):589–99. doi: 10.1016/j.chom.2017.10.005 29120742

[9] Blaxter M, Mann J, Chapman T, Thomas F, Whitton C, Floyd R, et al. Defining operational taxonomic units using DNA barcode data. Philos Trans R Soc Lond B Biol Sci 2005;360(1462):1935–43. doi: 10.1098/rstb.2005.1725 16214751 PMC1609233

[10] Schloss PD, Handelsman J. Introducing DOTUR, a computer program for defining operational taxonomic units and estimating species richness. Appl Environ Microbiol 2005;71(3):1501–6. doi: 10.1128/AEM.71.3.1501-1506.2005 15746353 PMC1065144

[11] Callahan BJ, McMurdie PJ, Holmes SP. Exact sequence variants should replace operational taxonomic units in marker-gene data analysis. ISME J 2017;11(12):2639–43. doi: 10.1038/ismej.2017.119 28731476 PMC5702726

[12] Callahan BJ, Wong J, Heiner C, Oh S, Theriot CM, Gulati AS, et al. High-throughput amplicon sequencing of the full-length 16S rRNA gene with single-nucleotide resolution. Nucleic Acids Res 2019;47(18):e103. doi: 10.1093/nar/gkz569 31269198 PMC6765137

[13] Gloor GB, Macklaim JM, Pawlowsky-Glahn V, Egozcue JJ. Microbiome datasets are compositional: And this is not optional. Front Microbiol. 2017;8:2224. doi: 10.3389/fmicb.2017.02224 29187837 PMC5695134

[14] Aitchison J. The statistical analysis of compositional data. J R Stat Soc Ser B: Stat Methodol 1982;44(2):139–60. doi: 10.1111/j.2517-6161.1982.tb01195.x

[15] Aitchison J. The statistical analysis of compositional data: Monographs on statistics and applied probability. London: Chapman & Hall Ltd.; 1986. 416 p.

[16] Egozcue JJ, et al. Isometric logratio transformations for compositional data analysis. Math Geology 2003;35(3): 279–300.

[17] Tu W. Zero-inflated data. Encyclopedia of environmetrics; 2006.

[18] Chen L, Reeve J, Zhang L, Huang S, Wang X, Chen J. GMPR: A robust normalization method for zero-inflated count data with application to microbiome sequencing data. PeerJ. 2018;6:e4600. doi: 10.7717/peerj.4600 29629248 PMC5885979

[19] Palarea-Albaladejo J, Martín-Fernández JA. Compositions — R package for multivariate imputation of left-censored data under a compositional approach. Chemometr Intell Lab Syst. 2015;143:85–96. doi: 10.1016/j.chemolab.2015.02.019

[20] Kim K, Park J, Jung S. Principal component analysis for zero-inflated compositional data. Comput Stat Data Anal. 2024;198:107989. doi: 10.1016/j.csda.2024.107989

[21] Tang Z-Z, Chen G. Zero-inflated generalized Dirichlet multinomial regression model for microbiome compositional data analysis. Biostatistics 2019;20(4):698–713. doi: 10.1093/biostatistics/kxy025 29939212 PMC7410344

[22] Ha MJ, Kim J, Galloway-Peña J, Do K-A, Peterson CB. Compositional zero-inflated network estimation for microbiome data. BMC Bioinform. 2020;21(Suppl 21):581. doi: 10.1186/s12859-020-03911-w 33371887 PMC7768662

[23] Zhang X, Guo B, Yi N. Zero-inflated Gaussian mixed models for analyzing longitudinal microbiome data. PLoS One 2020;15(11):e0242073. doi: 10.1371/journal.pone.0242073 33166356 PMC7652264

[24] Xu L, Paterson AD, Turpin W, Xu W. Assessment and selection of competing models for zero-inflated microbiome data. PLoS One 2015;10(7):e0129606. doi: 10.1371/journal.pone.0129606 26148172 PMC4493133

[25] Mardia KV, Jupp PE. Directional statistics. John Wiley & Sons; 2009.

[26] Scealy JL, Welsh AH. Regression for compositional data by using distributions defined on the hypersphere. J R Stat Soc Ser B: Stat Methodol 2011;73(3):351–75. doi: 10.1111/j.1467-9868.2010.00766.x

[27] Scealy JL, Welsh AH. Fitting Kent models to compositional data with small concentration. Stat Comput 2012;24(2):165–79. doi: 10.1007/s11222-012-9361-5

[28] Fletcher PT, Lu C, Pizer SM, Joshi S. Principal geodesic analysis for the study of nonlinear statistics of shape. IEEE Trans Med Imaging 2004;23(8):995–1005. doi: 10.1109/TMI.2004.831793 15338733

[29] Pennec X, Sommer S, Fletcher T., editors. Riemannian geometric statistics in medical image analysis. Academic Press; 2019.

[30] Borah K, Das HS, Seth S, Mallick K, Rahaman Z, Mallik S. A review on advancements in feature selection and feature extraction for high-dimensional NGS data analysis. Funct Integr Genomics 2024;24(5):139. doi: 10.1007/s10142-024-01415-x 39158621

[31] Li H. Microbiome, metagenomics, and high-dimensional compositional data analysis. Annu Rev Stat Appl 2015;2(1):73–94. doi: 10.1146/annurev-statistics-010814-020351

[32] Ju F, Zhang T. 16S rRNA gene high-throughput sequencing data mining of microbial diversity and interactions. Appl Microbiol Biotechnol 2015;99(10):4119–29. doi: 10.1007/s00253-015-6536-y 25808518

[33] Sharma A, Vans E, Shigemizu D, Boroevich KA, Tsunoda T. DeepInsight: A methodology to transform a non-image data to an image for convolution neural network architecture. Sci Rep 2019;9(1):11399. doi: 10.1038/s41598-019-47765-6 31388036 PMC6684600

[34] Zhu Y, Brettin T, Xia F, Partin A, Shukla M, Yoo H, et al. Converting tabular data into images for deep learning with convolutional neural networks. Sci Rep 2021;11(1):11325. doi: 10.1038/s41598-021-90923-y 34059739 PMC8166880

[35] Papa E, Docktor M, Smillie C, Weber S, Preheim SP, Gevers D, et al. Non-invasive mapping of the gastrointestinal microbiota identifies children with inflammatory bowel disease. PLoS One 2012;7(6):e39242. doi: 10.1371/journal.pone.0039242 22768065 PMC3387146

[36] Akiba T, Sano Y, Yoshida M, Koyama M, Hiraoka Y. Optuna: A next-generation hyperparameter optimization framework. In: Proceedings of the 25th ACM SIGKDD international conference on knowledge discovery & data mining; 2019. p. 2623–31.

